# Presepsin levels and COVID-19 severity: a systematic review and meta-analysis

**DOI:** 10.1007/s10238-022-00936-8

**Published:** 2022-11-15

**Authors:** Matteo Guarino, Benedetta Perna, Martina Maritati, Francesca Remelli, Caterina Trevisan, Michele Domenico Spampinato, Anna Costanzini, Stefano Volpato, Carlo Contini, Roberto De Giorgio

**Affiliations:** 1 Department of Translational Medicine, St. Anna University Hospital of Ferrara, University Ferrara, Via A. Moro, 44124, Ferrara, Italy; 2 Infectious and Dermatology Diseases, St. Anna University Hospital of Ferrara, University of Ferrara, Ferrara, Italy; 3 Department of Medical Sciences, St. Anna University Hospital of Ferrara, University of Ferrara, Ferrara, Italy

**Keywords:** COVID-19, Disease severity, Presepsin, SARS-CoV-2

## Abstract

Plasmatic presepsin (PSP) is a novel biomarker reported to be useful for sepsis diagnosis and prognosis. During the pandemic, only few studies highlighted a possible correlation between PSP and COVID-19 severity, but results remain inconsistent. The present study aims to establish the correlation between PSP and COVID-19 severity. English-language papers assessing a correlation between COVID-19 and PSP from MEDLINE, PubMed, Google Scholar, Cochrane Library, MeSH, LitCovid NLM, EMBASE, CINAHL Plus and the World Health Organization (WHO) website, published from January 2020 were considered with no publication date limitations. Two independent reviewers performed data abstraction and quality assessment, and one reviewer resolved inconsistencies. The protocol was registered on PROSPERO (CRD42022325971).Fifteen articles met our eligibility criteria. The aggregate study population included 1373 COVID-19 patients who had undergone a PSP assessment. The random-effect meta-analysis was performed in 7 out of 15 selected studies, considering only those reporting the mean PSP levels in low- and high-severity cases (*n* = 707).The results showed that the pooled mean difference of PSP levels between high- and low-severity COVID-19 patients was 441.70 pg/ml (95%CI: 150.40–732.99 pg/ml).Our data show that presepsin is a promising biomarker that can express COVID-19 severity.

**Supplementary Information:**

The online version contains supplementary material available at 10.1007/s10238-022-00936-8.

## Background

At the end of December 2019, a new zoonotic Coronavirus (SARS-CoV-2) was identified as the agent causing a cluster of pneumonia cases in Wuhan, China, and rapidly spreading throughout the world. Globally, data indicate that the COVID-19 pandemic involved over 530 millions of affected people with different clinical presentations and caused 6.3 millions of deaths [1, 2]. In February 2020, the World Health Organization designated the disease COVID-19 (coronavirus disease 2019), and different rates of mortality have been reported [3, 4]. Although many hypotheses have been proposed about its origin, the direct ancestral virus has not been identified yet [5, 6].

The clinical features of COVID-19 range from asymptomatic condition to severe/fatal lung injury and multi-organ failure due to an excessive immune response. Several risk factors for COVID-19 severity have been identified, namely a) “life-style factors” (e.g., obesity and smoking habit); b) demographic factors (e.g., age, male gender, post-menopausal status); and c) comorbidities (e.g., hypertension, coronary artery disease (CAD), diabetes, cerebrovascular disease (CVD), chronic kidney disease (CKD) and chronic obstructive pulmonary disease (COPD) [7]. Common complications of COVID-19 include acute respiratory distress syndrome (ARDS), acute kidney and liver dysfunctions, delirium/encephalopathy, thrombosis and cardiac damage (e.g., cardiomyopathy, arrhythmias and sudden cardiac death) [1].

Despite remarkable findings have been achieved since the beginning of the pandemic, an early identification and management of this novel coronavirus related disease is still limited. Since patients affected by COVID-19 may rapidly worsen and no effective antiviral therapy for SARS-CoV-2 infection has been found yet, an early identification of patients’ severity (through an effective and valuable biochemical marker) is key to guide the intensity of care and guarantee cardiorespiratory function [8]. In this regard, many efforts have been devoted to researching easily accessible biomarkers predicting COVID-19 severity.

Plasmatic presepsin (PSP) is a soluble N-terminal fragment of the cluster of differentiation marker protein 14 (CD14) reported to be a novel biomarker in sepsis [9, 10]. Indeed, as a glycoprotein expressed on monocytes and macrophages, CD14 is a receptor for the lipopolysaccharide (LPS)-LPS binding protein complexes, which is able to activate a series of signal transduction pathways leading to systemic inflammatory response. So far, two distinct forms of CD14 have been characterized, i.e., a membrane-bound (mCD14) and a soluble CD14 (sCD14). The sCD14 plays an essential role in mediating the immune responses to LPS of CD14-negative cells, such as endothelial and epithelial cells. During inflammatory stress, sCD14 is cleaved by plasmatic proteases which generate a truncated form of 64 aminoacidic residues of 13 kDa referred to as sCD14 subtype (sCD14-ST) or PSP [11, 12]. Since 2015, several studies have shown that PSP is not only useful for sepsis diagnosis [11–13], but also predicts the severity of this condition [14, 15]. A recent research highlighted that sepsis and SARS-CoV-2 infection share many immunopathological and pathophysiological similarities [16]. Therefore, it was recently postulated that elevated levels of PSP could predict the outcome of patients with SARS-CoV-2 infection [17, 18]. The relationship between PSP and COVID-19 severity is known, although not well detailed and comprehensively evaluated [17–31]. Thus, we conducted a systematic review and meta-analysis aimed at establishing the role of PSP in assessing SARS-CoV-2 infection severity.

## Methods

### Systematic review and meta-analysis

This paper has been performed following the Preferred Reporting Items for Systematic Reviews and Meta-Analyses (PRISMA) [32] and Meta-analysis of Observational Studies in Epidemiology (MOOSE) guidelines and checklists [33]. The protocol was registered on PROSPERO (CRD42022325971).

#### Data sources and searches

A literature search for relevant documents was performed in the following sources: MEDLINE, PubMed, Google Scholar, Cochrane Library, MeSH, LitCovid NLM, EMBASE, CINAHL Plus, and the World Health Organization (WHO) website. Items published from January 2020 were considered. No publication date limitations have been established. The used search strategy included the following Medical Subject Heading terms and keywords: (“Coronavirus” OR “Coronaviridae” OR “nCoV” OR “Coronavirus Infections” OR “COVID-19” OR “severe acute respiratory syndrome coronavirus 2” AND (“human presepsin protein” OR “Presepsin” OR “Plasmatic presepsin” OR “PSP” OR “sCD14-ST”). Only studies that involved humans, and were written in English, were included.

#### Study selection

**Fig. 1:**
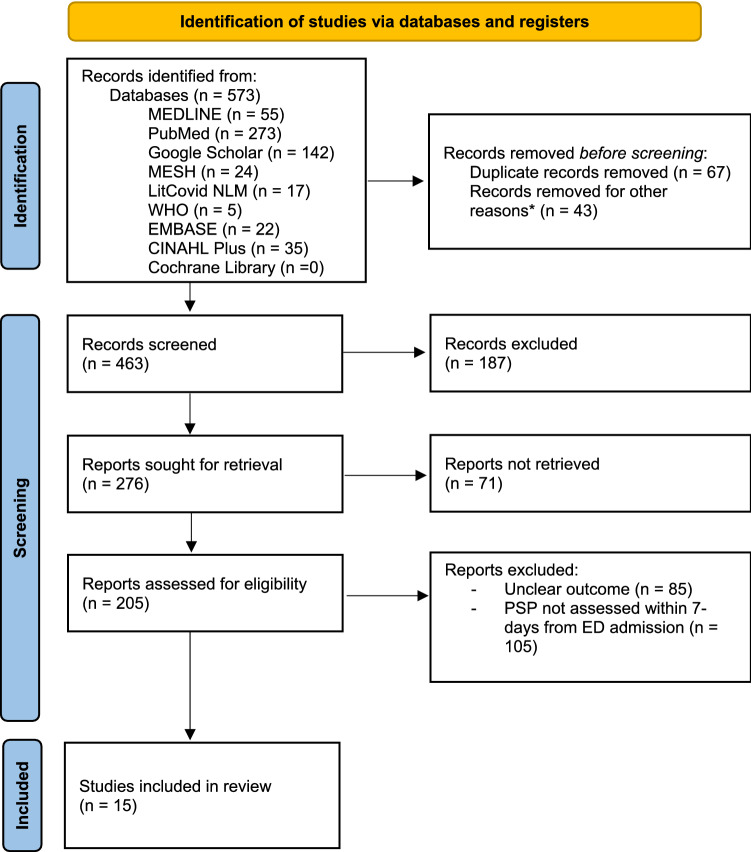
PRISMA 2020 flowchart for new systematic reviews that included only database and registry searches

The systematic review was performed comprehending prospective and retrospective studies, pooled analysis, cross-sectional studies and case series. A study was eligible for inclusion in this review if: (a) participants were affected by SARS-CoV-2 infection, confirmed through polymerase chain reaction testing of nasopharyngeal swab; (b) PSP levels were assessed within 7 days from the admission to the Emergency Department; (c) severe COVID-19 was defined as follows: SpO2 < 94% on room air and/or PaO2/FiO2 < 300 and/or respiratory rate > 30 breaths/minute and/or lung infiltrates > 50% [8–34]; (d) the outcome was measured in terms of mechanical ventilation requirement or intensive care admission or mortality; (e) correlation between PSP levels and disease severity was assessed (see Fig. 1 for recruitment and exclusion criteria).

The meta-analysis was performed comparing studies which expressed mean PSP levels, standard deviation (SD) and number of patients in two subgroups: experimental group (i.e., high-severity SARS-CoV-2 infection) *vs*. control group (i.e., low-severity disease).

Two independent reviewers (MG and BP) screened blindly the titles and abstracts of the identified documents and, for the record selected at this first step, retrieved and evaluated full manuscripts and appendices. Disagreements and inconsistencies were resolved by consensus and arbitration with a third reviewer (FR).

#### Data extraction and quality assessment

Two investigators (MG and BP) independently abstracted and recorded data, using standardized data abstraction form (Excel spreadsheet). The researchers were blinded to each other decisions. Extracted data included: study duration; study design; mean age; sex; sample size; numerosity of the two subgroups (when available); mean and SD of PSP levels in experimental and control group; time of PSP assessment and mortality. We did not contact study authors if data pertaining PSP levels or disease severity were not recorded. The quality assessment of the included studies has been performed following the NIH Quality Assessment Tool for Observational Cohort and Cross-Sectional Studies [35]. Each study was evaluated according to a standardized set of predefined criteria consisting of 14 items, mainly exploring the following domains: study population, exposure and outcome (Table 2). Each item was rated as positive, negative or not available. Two independent reviewers scored each article for quality and any scoring inconsistencies were resolved by discussion and consensus between the two reviewers.

#### Data synthesis and analysis

The meta-analysis was performed using the *meta* package of R statistical program (version 4.0.5) [36]. Mean serum levels of PSP and Standard Deviation (SD) in high- and low-severity patient groups were collected from the 7 out of 15 selected studies according to the data availability. Through a random-effect meta-analysis was performed to estimate the pooled mean difference and 95% confidence interval (95%CI) of serum levels of PSP between the high and low severity patients. Statistical heterogeneity was evaluated through Chi-squared test and expressed as I^2^ statistic of the proportion of total variation. A *p* value < 0.10 was considered statistically significant, and an I^2^ statistic > 75% indicated a high grade of heterogeneity. The publication bias of the selected studies was assessed both graphically and quantitatively, through test for asymmetry of funnel plots and Egger’s regression test, respectively. As a sensitivity analysis, we repeated our analysis after excluding those studies that could determine publication bias in light of the graphical evaluation of the funnel plot.

#### Role of the funding source

No funding sources have been used to produce this manuscript.

## Results

### Literature search results

A total of 573 studies were identified through database searching (273 from PubMed, 55 from MEDLINE, 24 from MESH, 22 from EMBASE, 35 from CINAHL, 142 from Google Scholar, 17 from LitCovid NLM, and 5 from WHO website). The flowchart of the studies’ selection is illustrated in Fig. 1. After the title-abstract and full-text screenings, 15 documents were identified, and their main characteristics are reported in Table 1. Among the selected studies, 14 involved adult (n = 1373) and one pediatric (n = 20) patients. All studies highlighted a possible relationship between PSP levels and COVID-19 severity, but this correlation was statistically significant in 10 works (1005 patients over 1373) [18, 19, 22–24, 27–31]. Among studies involving adult patients [17–25, 27–31], the mean age of the pooled sample was 62.7 ± 5.7 years. Mortality in the included studies ranged between 8 and 45%.

**Table 1:** Synopsis of the main features described in papers (n = 15) correlating PSP and COVD-19

Author	Year	Duration (months)	Study design	Patients (n)	Mean age (ys)	Male (n)	Level of PSP in low-severity patients (pg/ml)	Level of PSP in high-severity patients (pg/ml)	*p*	Time of PSP assessing	Mortality (%)	Conclusion
Fukada et al.[17]	2020	2	Case series	6	N/A	N/A	N/A	N/A	N/A	Admission	16.0	PSP has potential as a biomarker for severe COVID-19 pneumonia
Zaninotto et al.[18]	2020	3	Retrospective study	75	67.0	56	408	1069	< 0.001	Day 2–7	8.0	PSP seems to have a role in providing prognostic information in COVID-19 pts in the early phase
Dell'Aquila et al.[19]	2020	2	Prospective study	143	73.0	86	518	892	< 0.001	Admission	45.0	PSP is a very specific predictor (92%) of 30-day mortality in COVID-19 patients
Ducastel et al.[20]	2020	2	Retrospective study	160	60.0	92	N/A	N/A	N/A	Admission	10.6	PSP was associated with worse outcome
Keskinidou et al.[21]	2020	8	Observational study	66	64.0	51	N/A	1300	N/A	Within 24 h post ICU admission	34.9	PSP could differentiate patients who did not survive, independently of dexamethasone administration
Schirinzi et al.[22]	2020	2	Observational study	86	67.0	58	737	1234	< 0.0001	Admission (and every 24 h)	22.0	PSP reflects the clinical course of the disease and might be used to predict the evolution of COVID-19 disease
Kocyigit et al.[23]	2020	2	Observational study	88	51.0	41	590	3500	< 0.001	Within 24 h post admission	N/A	There was a significant correlation between PSP and disease severity
Hasegawa et al. [24]	2021	1	Observational study	57	59.0	33	563	1217	0.007	Admission	N/A	PSP is significantly higher in COVID-19 pts with ARDS
Domi et al.[25]	2021	11	Retrospective study	97	68.0	67	433	579	0.183	Admission	14.4	The complication of bacterial superinfection might be associated with PSP elevation
Dewi et al.[26]	2021	8	Cross-sectional study	20	10.0	10	N/A	N/A	N/A	Admission	40.0	High levels of PSP were related to higher mortality rate
Mirza et al.[27]	2021	10	Cross-sectional study	80	67.5	54	17	56	< 0.001	Admission	N/A	PSP is the most useful tool in predicting the severity of COVID-19 infection
Farag et al.[28]	2021	4	Observational study	42	59.6	26	390	950	0.008	Admission	26.2	Potential utility of PSP as a predictive indicator of severity in COVID-19 patients
Kim et al.[29]	2021	N/A	Retrospective study	42	59.5	20	N/A	N/A	0.007	Admission	N/A	PSP has the potential to be a useful severity marker in patients with COVID-19
Çaglar et al.[30]	2022	3	Observational study	259	58.1	146	40.17	55.40	0.013	Admission	14	Presepsin may be of value for risk stratification of COVID-19 patients
Morales-Cely et al. [31]	2022	N/A	Prospective study	152	N/A	N/A	570	1358	< 0.0001	Admission	N/A	Median level of PSP was higher in patients deceased by COVID-19 than in survived

N/A: not available; PSP: Plasmatic presepsin

Concerning the study design, we selected 5 observational, 4 retrospective, and 2 prospective studies, 2 cross-sectional works, one case series and one pooled analysis. The quality assessment is reported in Table 2. No study was excluded because of a quality score less than 8 (< 50%).

**Table 2:** NIH Quality Assessment Tool for Observational Cohort and Cross-Sectional Studies

Authors	Year	Questions														Quality Rating
		1	2	3	4	5	6	7	8	9	10	11	12	13	14	
Fukada *et al.*[17]	2020	Y	Y	N/A	Y	N	Y	N/A	N/A	Y	Y	N	N	N	N	Poor
Zaninotto et al. [18]	2020	Y	Y	N/A	Y	N	Y	Y	N/A	N	Y	Y	N	Y	N	Fair
Dell'Aquila et al.[19]	2020	Y	Y	Y	Y	Y	Y	Y	N/A	Y	Y	Y	N	Y	Y	Good
Ducastel et al.[20]	2020	Y	Y	Y	Y	N	Y	Y	N/A	Y	N	Y	N	Y	N	Good
Keskinidou et al.[21]	2020	Y	Y	Y	Y	N	Y	Y	N/A	Y	N	Y	N	Y	N	Fair
Schirinzi et al.[22]	2020	Y	Y	Y	Y	Y	Y	Y	N/A	Y	N	Y	N	Y	N	Good
Kocyigit et al.[23]	2020	Y	Y	Y	Y	Y	Y	Y	N/A	Y	N	Y	N	Y	Y	Good
Hasegawa et al.[24]	2021	Y	Y	Y	Y	N	Y	Y	N/A	Y	N	Y	N	Y	N	Fair
Domi et al.[25]	2021	Y	Y	Y	Y	Y	Y	Y	N/A	Y	N	Y	N	Y	N	Good
Dewi et al.[26]	2021	Y	Y	N	Y	N	Y	N/A	N/A	Y	Y	Y	N	Y	N	Fair
Mirza et al.[27]	2021	Y	Y	Y	Y	Y	Y	Y	N/A	Y	N	N	N	Y	N	Fair
Farag et al.[28]	2021	Y	Y	Y	Y	Y	Y	Y	N/A	N	Y	N	N	Y	Y	Good
Kim et al.[29]	2021	Y	Y	Y	Y	Y	Y	N/A	N/A	N	Y	Y	N	N/A	N	Fair
Çaglar et al.[30]	2022	Y	Y	Y	Y	Y	Y	Y	N/A	Y	N	Y	N	Y	Y	Good
Morales-Cely et al.[31]	2022	Y	Y	N/A	Y	Y	Y	N/A	N/A	N	N	Y	N	N/A	N	Poor

*Note:* N/A = Not applicable; Y = Yes; N = No

Questions

Was the research question or objective in this paper clearly stated?

Was the study population clearly specified and defined?

Was the participation rate of eligible persons at least 50%?

Were all the subjects selected or recruited from the same or similar populations (including the same time period)? Were inclusion and exclusion criteria for being in the study prespecified and applied uniformly to all participants?

Was a sample size justification, power description, or variance and effect estimates provided?

For the analyses in this paper, were the exposure(s) of interest measured prior to the outcome(s) being measured?

Was the timeframe sufficient so that one could reasonably expect to see an association between exposure and outcome if it existed?

For exposures that can vary in amount or level, did the study examine different levels of the exposure as related to the outcome (e.g., categories of exposure, or exposure measured as continuous variable)?

Were the exposure measures (independent variables) clearly defined, valid, reliable, and implemented consistently across all study participants?

Was the exposure(s) assessed more than once over time?

Were the outcome measures (dependent variables) clearly defined, valid, reliable, and implemented consistently across all study participants?

Were the outcome assessors blinded to the exposure status of participants? Was loss to follow-up after baseline 20% or less?

Were key potential confounding variables measured and adjusted statistically for their impact on the relationship between exposure(s) and outcome(s)?

## Study characteristics

The characteristics of the studies with main clinical features eligible for our paper are summarized in Table 1. The quality assessment has been performed following NIH criteria (Table 2). No study was excluded solely because of low-quality scores less than 8 (< 50%). No randomized, controlled trials met our selection criteria. This manuscript involved observational (*n *= 6), retrospective (*n* = 4) and prospective (*n* = 2) studies, cross-sectional analysis (*n* = 2) and case series (*n* = 1).

## Publication bias and heterogeneity

The evaluation of the funnel plot suggested a possible publication bias across the selected studies (Supplementary Fig. 1), which was not confirmed at the Egger’s regression test (Egger regression intercept = 3.558, 95%CI: -0.376, 7.492, *p* = 0.130). In order to establish the level of consistency among involved studies, the heterogeneity has been calculated, resulting quite high (I^2^ = 93%, *p* < 0.01).

## Serum level of PSP and clinical outcomes

**Fig. 2:**
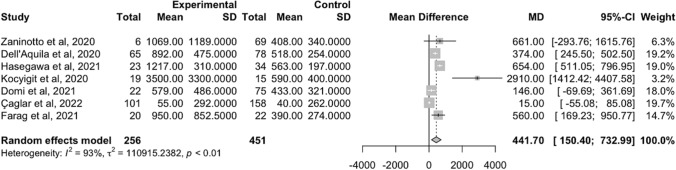
Forest plot on the mean difference of PSP levels between the high- and low-severity patient groups from the random-effect meta-analysis

Among the selected studies, higher serum levels of PSP at hospital admission in patients with COVID-19 disease were related to worse clinical outcomes (i.e., higher severity of COVID-19 and necessity of respiratory support). The random-effect meta-analysis on 707 individuals (Fig. 2) showed a significant pooled mean difference in serum PSP levels between patients with high- and low-severity of COVID-19 disease was 441.70 pg/ml (95%CI: 150.40–732.99 pg/ml).

## Sensitivity analysis

The results were confirmed in the sensitivity analysis after excluding the study of Kocyigit et al. [23]. Indeed, we excluded the analysis with more graphical distance of the effect size from the polled one in the forest plot of meta-analysis and with the smallest sample size. From the additional meta-analysis, including 673 patients, we found a pooled mean difference of PSP between the high- and low-severity patient groups of 350.02 pg/ml (95%CI: 115.15–584.89 pg/ml) (Supplementary Fig. 2).

## Discussion

Early prediction of COVID-19 severity is still challenging although it represents a crucial step in defining the risk of fatal outcomes and the most appropriate recovery setting for adequate treatment. As reported by the last update of the Surviving Sepsis Campaign Guidelines [8], disease severity, which is currently assessed mainly by clinical parameters, has a key role in managing COVID-19 patients. However, since these criteria do not predict the risk of clinical worsening, a tool able to assess COVID-19 evolution would be helpful for physicians. Furthermore, it is not currently possible to define the severity of the disease relying upon the viral load [34].

Different biochemical markers have been proposed to integrate the WHO criteria in predicting COVID-19 severity. In particular, a recent research performed on diabetic patients confirmed that C-reactive protein (CRP) is a valuable predictor of COVID-19 progression and severity. Furthermore, serum levels of inflammation-related (e.g., interleukin-6 or serum ferritin) and coagulation parameter (D-dimer) were higher in patients with SARS-CoV-2 infection and diabetes mellitus *vs*. those without, suggesting that diabetic patients could be more susceptible to the cytokine storm that leads to ARDS and fatal outcome [37]. However, different factors can alter levels of these markers (e.g., tumors, autoimmune diseases) making them less specific in the diagnosis and risk stratification of patients with COVID-19 [38].

Recently, different studies highlighted the role of a novel biochemical marker (i.e., PSP), which seems to have better sensitivity and specificity in the diagnosis and severity assessment of sepsis [9–15, 37]. Considering that sepsis and SARS-CoV-2 infection share immunopathogenetic and pathophysiological similarities, we believe that PSP may help in risk stratification [16]. In the last two years of pandemic, the interest in the possible correlation between COVID-19 severity and levels of PSP has increased and several studies have been published [17–31]. In 2021, Amhed et al. proposed a review on this correlation [39] highlighting that PSP levels predicted the aggravation of COVID-19 infection. However, the limited number of pertinent manuscripts hampered this analysis as only three articles [17, 18, 22] were considered eligible for the review.

In 2021, Lippi et al*.* proposed a pooled analysis on this topic concluding that PSP values were significantly higher in COVID-19 patients with severe/critical illness *vs*. those without [24]. In our opinion, this result was interesting but the sample size was small (*n* = 420). Moreover, this paper presented an unclear definition of disease severity (i.e., death, need for tracheostomy, mechanical ventilation, respiratory distress, ICU recovery).

Our work considered studies that assessed PSP levels in the first 7 days of hospitalization. This choice allowed us to consider, not only patients identified as critical because of their clinical manifestations, but also those ones who showed a rapid worsening in the first days from admission. Indeed, Faes et al. reported that an average time of 5 to 7 days to progress from the first manifestations to ARDS [40]. As indicated by the pooled results, PSP can be considered a valuable biomarker of COVID-19 severity. Indeed, higher PSP levels might help physicians in recognizing potentially critical patients, even when clinical condition are not alarming yet.

We would like to acknowledge some limitations of our study: First, there is a complete lack of multicenter randomized clinical trials, which are fundamental to confirm the effective usefulness of this biomarker to stratify COVID-19 severity. Second, the heterogeneity among the involved studies resulted very high (> 90%). This result was expected since PSP has only recently been proposed as a biomarker for COVID-19 severity with few published studies. Third, all the included studies in the present meta-analysis considered the disease severity as a primary outcome. The definition of this condition is often heterogeneous; therefore, the choice of other outcomes (e.g., mortality) might be preferable. Fourth, the included studies used different PSP assessment methods (e.g., PATHFAST Presepsin–Mitsubishi Chemical Europe GmbH, Düsseldorf, Germany or STACIA Presepsin–LSI Medience Corporation, Tokyo, Japan), which can be a further source of heterogeneity.

Main strengths of this study include: First, we performed a systematic review on the role of PSP in COVID-19 severity according to specific guidelines. Second, we examined a significant number of scientific databases leading to a consistent number of eligible papers (resulting in a large sample size). Third, this study allowed for a new quantitative analysis on this topic.

## Conclusion

Our results show that PSP alone is a reliable tool to assess COVID-19 severity. The possible integration of this biomarker with clinical criteria might be useful to improve the accuracy of risk stratification in COVID-19 patients. Furthermore, since SARS-COV-2 infection and sepsis share similar immunopathological manifestations and PSP showed its intrinsic value in predicting the severity of both diseases, we can hypothesize that other conditions with similar immunopathological features, a biomarker as PSP might help in the risk stratification of affected patients.

## Supplementary Information

Below is the link to the electronic supplementary material.

Supplementary file1 (DOCX 110 kb)

Supplementary file2 (DOCX 220 kb)

## Data Availability

The dataset is available for reviewers on reasonable request.

## Declarations

### Reproducible research statement

The study protocol was registered on PROSPERO (CRD42022325971).

### Statistical code and data set

All data and statistical analyses codes are available upon request to the corresponding author.
