# Supplementary figures and images for: Presepsin levels and COVID-19 severity: a systematic review and meta-analysis
Source: Clin Exp Med. 2022 Nov 15;23(4):993–1002. doi: 10.1007/s10238-022-00936-8 (PMC9666937; doi:10.1007/s10238-022-00936-8)

**Supplementary Figure 1.** Funnel plot of the included studies.


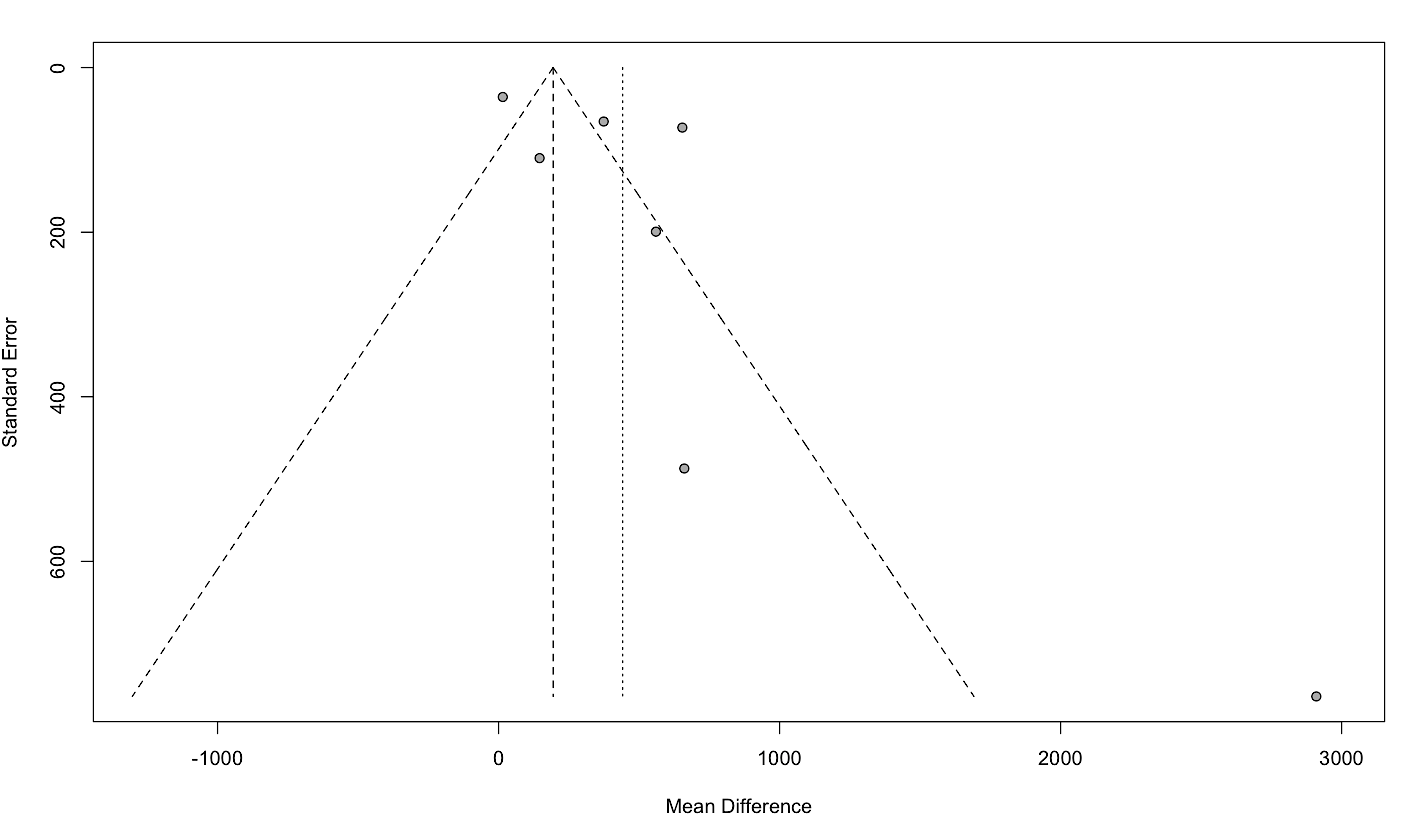

Supplement: Supplementary file 1 — Supplementary file1 (DOCX 110 kb) [file 10238_2022_936_MOESM1_ESM.docx]

**Supplementary Figure 2.** Forest plot after excluding the study of Kocyigit *et al*.


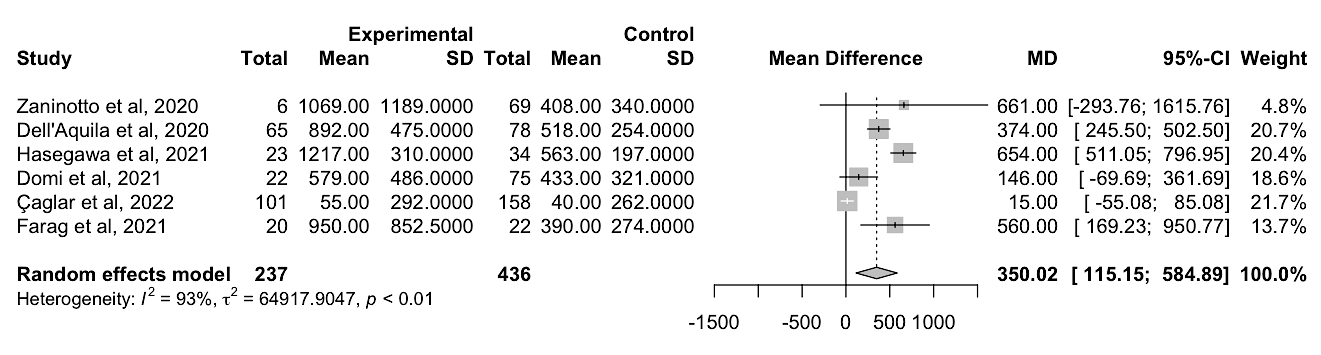

Supplement: Supplementary file 2 — Supplementary file2 (DOCX 220 kb) [file 10238_2022_936_MOESM2_ESM.docx]
